# Micromorphology and native extractive behaviour of wood powder

**DOI:** 10.1038/s41598-024-75716-3

**Published:** 2024-10-26

**Authors:** Atanu Kumar Das, David A. Agar, Magnus Rudolfsson, Petri Kilpeläinen, Jenni Tienaho, Dinesh Fernando

**Affiliations:** 1 Department of Forest Biomaterials and Technology, Swedish University of Agricultural Sciences, Umeå, SE-901 83 Sweden; 2 Cellulose Technology, Department of Sustainable Materials and Packaging, MoRe Research AB, RISE Research Institutes of Sweden, Hörneborgsvägen 10, Domsjö, Örnsköldsvik, 892 50 Sweden; 3 Natural Resources Institute Finland (Luke), Production systems, Latokartanonkaari 9, Helsinki, FI-00790 Finland; 4 Department of Forest Biomaterials and Technology/Wood Science, Swedish University of Agricultural Sciences, Uppsala, SE-756 51 Sweden

**Keywords:** Milling techniques, Cytochemical staining, Drying, Extractive distribution, Energy science and technology, Materials science

## Abstract

The transition to a bioeconomy is attracting the use of wood powders for developing bio-based chemicals, fuels, and products to replace fossil-based products. Wood powder-based products depend on the properties and quality of wood powders. Despite many studies on their morphological and physical properties, studies on micromorphology and extractive micro-distribution are scarce. Here we investigated the effect of milling type and wood quality in terms of moisture content on microstructural changes and native extractive distribution in wood powders. The findings showed that non-dried and dried multi-blade shaft mill (MBSM) powders had smooth surfaces and less undamaged cellulosic fibre walls, and extractives were located in the cell lumen. Non-dried and dried hammer mill powders had a rough surface and fibres with structural deformations in their cell walls (e.g. dislocations). Extractives were redistributed on the particle surface as well as dispersed in the cell lumen for both types of hammer mill powders. In a word, the powders obtained from MBSM technology are more native in structure. The findings of the study can have implications for downstream processes.

**Supplementary Information:**

The online version contains supplementary material available at 10.1038/s41598-024-75716-3.

## Introduction

Sustainable and circular bioeconomy is growing based on the implications of renewable biomaterials that can replace fossil-based feedstocks. Wood, a renewable biomaterial, plays a significant role in the phase-out of the fossil-based economy^1,2^. Thus, it works as carbon neutrality^3,4^. However, wood size needs to be reduced for the applications in downstream processes. Wood powders have versatile industrial applications, i.e., bio-based chemicals^5,6^, 3D printing^7^, paperboard production^8^, paper making^9^, wood pellet production^10^, and wood-based composites (wood plastic composites (WPC) and particleboard)^11,12^.

Wood powder properties have a vital influence on downstream applications. There have been many studies on the morphology of wood powders, i.e., size, shape, surface area, and porosity^13^. The roughness of powder is also analysed, and it causes the bridging issue leading to handling problem^14^. Particle size < 1 mm is required for optimal combustion efficiency during co-firing of wood powders^15^. The carbon yield in char depends on the particle size^16^, which controls the heating and reaction rates^17^ during pyrolysis. Smaller particles suppress the char-forming reactions and reduce the vapour-phase residence time^18^, leading to high bio-oil^19^ and tar^17^ production.

The mechanical stress on wood causes the dislocations of the cell wall^20^. Dislocation changes the microfibril direction of the fibre cell wall resulting a deviation of microfibril angle (MFA) from the surrounding cell wall^21,22^. Thus, it may change the crystalline structure of the cellulose fibrils. Consequently, the low crystallinity enhances the conversion of cellulose to glucose by hydrolysis^5^, and it decreases with the decreasing of particle size^23^. On the other hand, the high crystallinity results in high mechanical properties of the composite, i.e., paper^24^ and it provides high thermal resistivity of the composite, i.e., nanocellulose-based nanocomposite^25^.

Wood powder production technologies influence the morphology of wood powder. For example, a two-stage hammer mill can produce < 1 mm powders^15^, while knife mills generate 1.5 mm powders^26^ from the pre-dried wood chip. The wood powders produced from hammer mills are elongated, and these are rectangular shapes for knife mills^14^. Wood powder surface is also affected by technologies and hammer mill powders are found to have a less rough surface than knife mill powders^14^.

The chemistry of wood powders, in particular extractive distribution also influences their applications in developing value-added products. The presence of extractives in particles reduces the properties of composite products by weakening the interfacial bonding between wood particles and the binding agent in the composite matrix^27,28^. Extractives also control the pelletisation efficiency. The low extractive content causes the high energy consumption to produce pellets^29^. The lubrication effect of extractive lowers the friction in die channels facilitating the pelletisation^30^. On the other hand, it reduces the bonding between particle to particle leading to less durable wood pellets^30^. Nielsen et al.^31^ have also observed that extractive removal increases the wood pellet strength properties.

However, the extractive distribution analysis in wood powders is a dark science. The distribution of wood extractives on the surface of wood powders may affect performance in different applications. It may influence the reactivity during chemical treatment. Presence of extractives in wood powders is reported to lower the adhesion properties between wood powder and polypropylene leading to lowering the strength properties of wood powder- polypropylene composite^32^. The authors have not considered the distribution of extractive in wood powder rather than the total amount of extractives. The interfacial bonding between adhesive and wood powder in the composite matrix may be affected depending on the distribution of extractives on the wood powder surface. The study on the extractive distribution on the wood powder surface may help explain the difference in the chemical reaction and bonding strength in the matrix for different powder types. However, there is limited studies analysing the native extractive distribution/redistribution of processed wood powders.

This study was conducted to investigate the morphology of wood powders and distribution/redistribution of native extractives in the wood powders obtained from Scots pine (*Pinus sylvestris* L.) wood by a prototype multi-blade shaft mill (MBSM). The effect of milling types and operation parameters on the wood powder morphology and extractive distribution in wood powders was considered. The behaviour of wood powder morphology and extractive distribution during their drying was also analysed in the study. Hammer mill powders obtained from a single setting were used for the comparison. Original wood samples were also analysed to determine the changes of extractive distribution in wood powders.

## Materials and methods

### Wood and wood powder samples

Samples from fresh Scots pine wood and their wood powders milled by a prototype multi-blade shaft mill (MBSM)^33^ were used in the current study. The plant collection and use were in accordance with all the relevant guidelines. Wood discs of 2 cm thick were collected from both ends of the wood trunk before milling. Powders of low, high, and medium bulk density were used for the study. The source of the wood disc and wood powders was from a similar wood trunk for each type. Sampling of wood powders was carried out after milling the wood and immediately taking them into an air-tight plastic bag. Never-dried wood powders and fresh wood discs were kept in the refrigerator at -20 °C immediately after sampling until microscopy analysis. Wood powders dried at 105 °C overnight were also used for analysing the effect of drying on morphology and extractive distribution. Dried powders in an air-tight plastic bag were kept at room temperature for further analyses. Hammer mill powders obtained from a single setting were used for comparison with MBSM powders. The process parameters applied during wood powder production and wood powder properties are shown in Table S1. The sampling stages of the wood disc and wood powders are presented in Fig. S1.

### Extractive analysis

This analysis was considered to investigate the effect of milling technology and wood powder drying on extractive content. Thus, it was designed to conduct analysis of some specific extractives. Extractive was extracted using a speed Extractor E-916 with four cycles. The solvent was 95% acetone, and the amount of sample was 2 g. The required amount of solvent was 50 ml. The solvent was removed by an evaporator, and the rest of water was removed by overnight drying at 30 ^o^C in the oven dryer. Extractives were then analysed with gas chromatography mass spectrometer (GS-MS) HP6890-5973 (Hewlett Packard, Palo Alto, CA, USA) instrument^34^. Heneicosanoic (C21:0) and betulinol were used as internal standards in analyses. Dried samples were dissolved in acetone (10 ml) and an aliquot (100 µl) of each sample were evaporated to dryness under nitrogen flow and further dried in a vacuum oven to dryness. Samples were then silylated by adding pyridine, N,O-bis(trimethylsilyl) trifluoroacetamide (BSTFA, Supelco Analytical, Bellefonte, PA, USA) and trimethylsilyl chloride (TMCS, Merck KGaA, Darmstadt, Germany). The silylated samples were then analysed by GC-MS and identified by NIST14/Wiley11 and laboratory’s own mass spectrometry libraries.

### Light microscopy

The localization and morphological features of major lipophilic extractives (e.g. triglyceride, total lipids, and unsaturated fats) and their spatial micro-distribution and redistribution in different wood powder types and during their drying, were investigated using light microscopy (LM) in combination with histochemical techniques. Wood powder morphology was also analysed using LM. For fresh wood samples, small wood blocks were cut from wood disks using a hand saw and ca. 10 μm thin sections were taken from the blocks using a sliding microtome for histochemical analysis with LM.

#### Micro-morphological analysis

Never-dried wood powder and dried wood powder samples were stained separately with 0.1% Saffranin (w/v) for ca. 3–5 min followed by rinsing with water to remove excess stain. Stained wood powder samples were mounted on glass slides with 1–2 drops of glycerol-gelatin, covered with cover slips and used for morphological analysis using LM. Stained samples on glass slides were examined using a Leica DMLB light microscope and Images recorded digitally using an Infinity X-32 camera (DeltaPix, Denmark). A Leica IM50 Image Manager was used for processing and storing the images.

#### Nile blue staining for triglyceride

Nile blue (NB) staining method was performed for all samples, i.e. fresh wood blocks, dried- and never-dried wood powders. All solutions necessary for the method, i.e., 1% aqueous NB (w/v), deionized water, and 1% acetic acid (v/v) were pre-warmed to 37 ^o^C. All samples (i.e. wood sections and powders) were then stained as follows. Samples were collected into small glass vials, immersed in the NB solution by adding a few drops on to the samples and heated at 37 ºC for 5 min. Samples were then washed quickly in warm water, differentiated in 1% acetic acid at 37 ºC for 30 s followed by three times washes in warm water (37 ^o^C). Stained samples were finally mounted in glycerol-gelatin on glass slides, covered with cover slips and used for LM. Imaging and Image analysis were done as described above.

#### Sudan black B for total lipid

Wood powders and tissue sections were processed as described above except the staining technique. Samples were immersed in 70% ethanol for few seconds, and after removing excess ethanol, the samples were then stained in saturated Sudan black B in 70% ethanol for 20 min followed by differentiation in 70% ethanol (ca. one min.) in order to remove any deposits of Sudan black. Subsequently, they were rinsed with 50% Ethanol and water and mounted in glycerol-gelatin on glass slides. Imaging of the stained samples, digital image recording and storing were carried out as described above.

#### Osmium tetroxide for unsaturated fat

Wood powders and tissue sections in small glass vials were immersed in a 1% w/v aqueous osmium tetroxide solution with agitation for proper mixing and kept for one hour at room temperature in a fume cupboard. Samples were then rinsed well with ultra-pure water, mounted in glycerol-gelatin and covered with cover slips before examining and imaging with LM.

### Micro-structural deformation

Micro-structural distortions within fibre walls due to different processing when making wood powders were analysed using polarized light microscopy (PLM). This allows visualization of such fibre cell wall deformations (e.g. dislocations) due to the birefringence properties of crystalline cellulose of the fibre wall^20,21^. Wood powders were processed as described above except any staining applied. The sample was soaked in water for five min, mounted on glass slides with glycerol-gelatin, covered with cover slip and examined under PLM (crossed polars; Leica DMLB, Infinity X-32 digital camera).

## Results and discussion

### Micromorphological properties

#### Effect of milling type and parameters and wood powders drying on surface and fibre properties

**Fig. 1:**
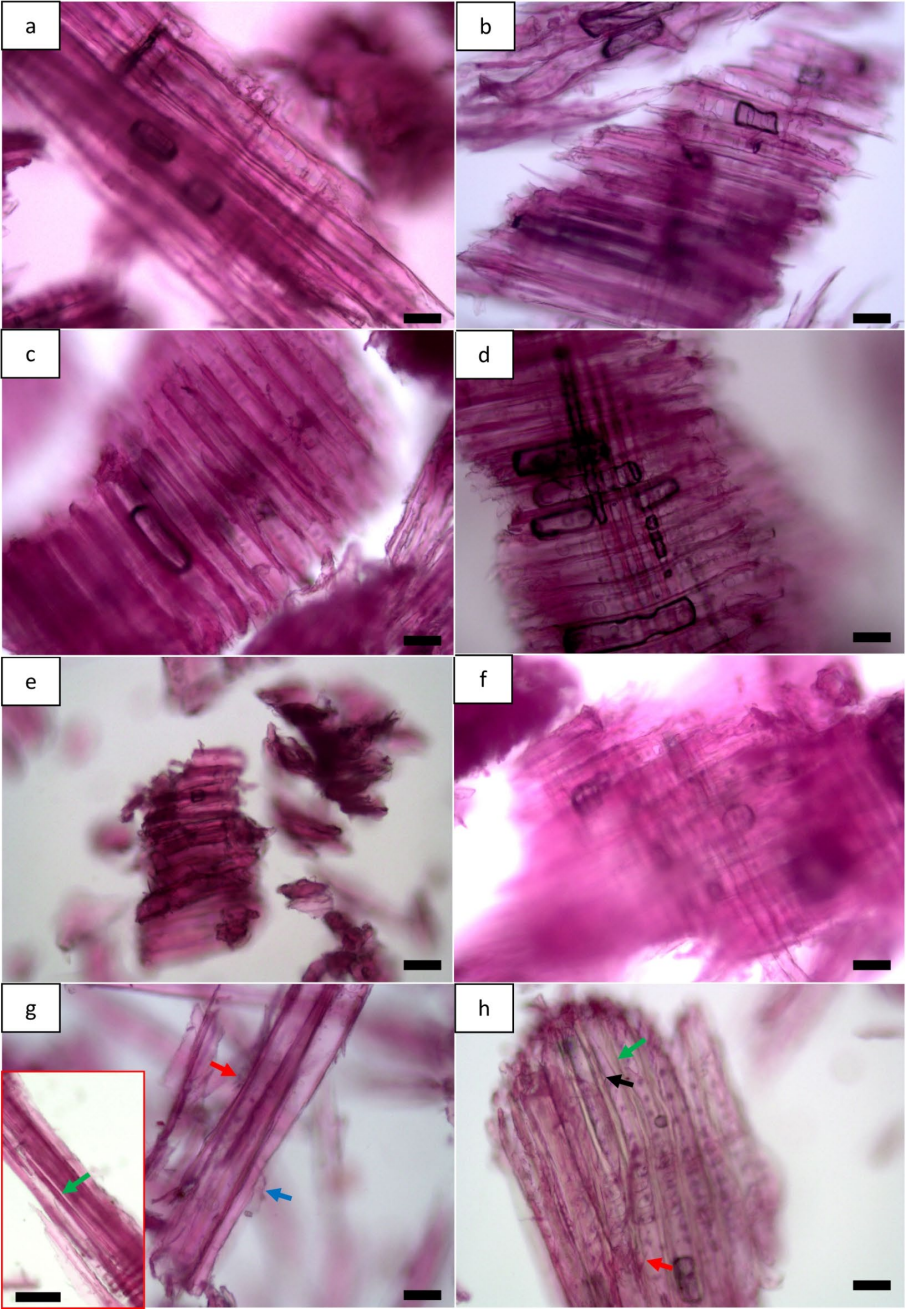
Light micrographs showing effects of milling types and parameters and wood powders drying on the surface and fibre properties for MBSM (**a-f**) and hammer mill (HMP) powders (**g**, **h**): (**a**,** c**,** e** and **g**) show non-dried MBSM and HM powders where (**a**) is green wood powder (GMP); (**c**) for fibre saturation wood powder (FMP); (**e**) for dry wood powder (DMP); and (**g**) for hammer mill powder (HMP). (**b**,** d**,** f** and **h**) show dried MBSM and HM powders where (**b**) for green wood MBSM powder (GMP); (**d**) for fibre saturation wood MBSM powder (FMP); (**f**) for dry wood MBSM powder (DMP); and (**h**) for hammer mill powder (HMP). Bars: a, b, c, d, e, f, g, h, 30 μm; g (inset bottom left), 100 μm.

The surface of non-dried and dried powders obtained from MBSM (powder from green wood (GMP), powder from fibre saturation wood (FMP), powder from dry wood (DMP)) was comparatively smooth (Fig. 1a-f), while the hammer mill produced powders (HMP) having rough surfaces (Fig. 1g and h). Further, less or no fibre defibration and fibrillation were observed for non-dried and dried GMP, FMP and DMP powders (Fig. 1a-f). In contrast, non-dried (Fig. 1g) and dried (Fig. 1h) HMP powders showed fibre defibration (green arrows) and fibrillation (red arrows). Peeling of fibrils from the fibre surface was observed for non-dried HMP powders (blue arrow, Fig. 1g) and fibre separation/release from wood particle (black arrow, Fig. 1h) was also observed for dried HMP powders. Observations thus indicated MBSM milling parameters had no impact on wood powder surfaces and fibres, but hammer mill had impact on powders with regards to surface roughness and fibre deformation/separation. The hammer mill reduces the wood size using impact and shear force, and the mechanism for MBSM is cutting or swing using sharp metal blades^33^. The different size reduction mechanisms may cause differing wood powder surfaces and fibre properties. Impact and shear force can damage fibres of wood powders more than cutting, resulting in fibre structural deformations with rough surfaces and fibre separation for HMP. The drying temperature of wood powders was 105 ºC that appears to have no much effect on the surface properties of wood powders.

The physical appearance of MBSM and hammer mill powders was investigated, and results indicated considerable differences between them. Fig. S2 shows appearance of the two major milled powders where MBSM powders (DMP as a reference powder; Fig. S2a) were more spherical compared to hammer mill powders (Fig. S2b). There is a stark difference in shape, i.e., sphericity, between them in which the majority of MBSM particles were more or less similar in appearance with close to spherical in shape while HMP was less spherical (particles with longer length than width) and very dissimilar in shape with varying both the length and the width of the particles. The cutting mechanism, i.e., uniform cutting given by the unique design of the multi-blade shaft milling may responsible for obtaining similar shape properties while non-uniform impact and shear force by mechanical attrition process from hammer mill presumably influenced the production of observed elongated powders with different shape.

#### Effect of milling type and parameters and wood powders drying on micro-structural deformation

**Fig. 2:**
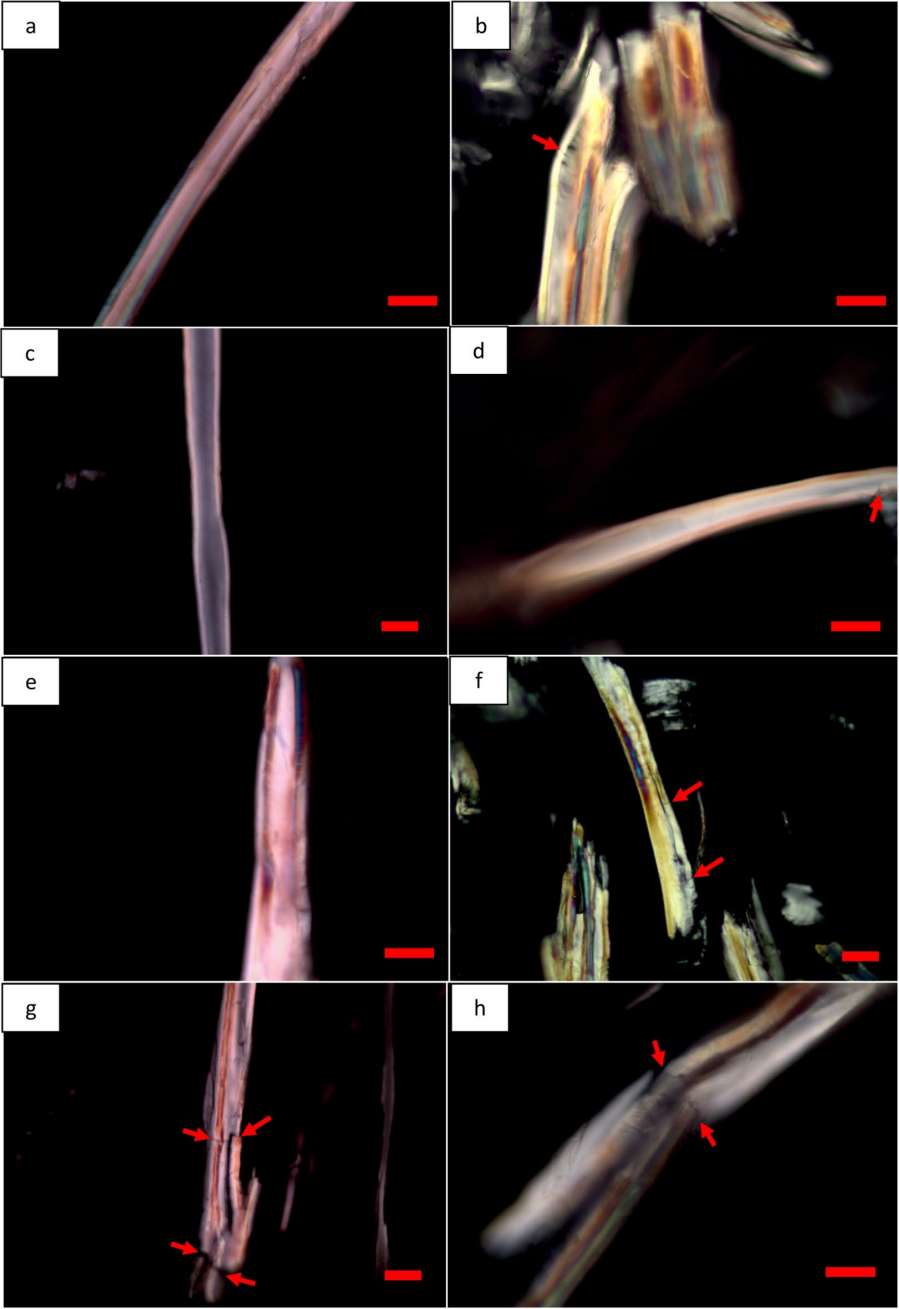
Milling types and parameters and wood powder drying effect on the micro-structural deformation for MBSM (**a-f**) and hammer mill (HMP) powders (**g**,** h**): (**a**,** c**,** e** and **g**) show non-dried MBSM and HM powders where (**a**) is green wood powder (GMP); (**c**) for fibre saturation wood powder (FMP); (**e**) for dry wood powder (DMP); and (**g**) for hammer mill powder (HMP). (**b**,** d**,** f** and **h**) show dried MBSM and HM powders where (**b**) for green wood MBSM powder (GMP), (**d**) for fibre saturation wood MBSM powder (FMP); (**f**) for dry wood MBSM powder (DMP); and (**h**) for hammer mill powder (HMP). Bars: a, b, d, e, h, 20 μm; c, f, g, 30 μm.

Fibre structural deformations (e.g. dislocations of fibre cell walls) in wood powders were also investigated using polarized light microscopy. The disrupted brightness (red arrow in Fig. 2b, d, f, g and h) indicated deformation/damages to the native crystalline structure of their cellulose fibrils. Fibre cell walls of non-dried MBSM powders (GMP, FMP, DMP) were found to be less damaged and/or preserved (Fig. 2a, c and e), but dried MBSM powders (red arrows in Fig. 2b, d and f) had some fibres having mild defects in their cell walls. However, both non-dried (red arrow, Fig. 2g) and dried (red arrow, Fig. 2h) HMP powders were found to contain considerable fibre wall defects (e.g. fibre wall breakage, dislocation etc.) presumably indicating presence of induced non-crystalline regions in their fibre walls. Impact mills, i.e., hammer mill use impact and shear mechanical stresses to reduce the size of biomass^35^. Therefore, the applied shear force on wood was severe for hammer mill than MBSM since MBSM applied cutting force by sharp blades. Consequently, the shear force disrupted/damaged the native structure of fibre cell walls for HMP powders while the milling parameters had no or less impact on crystalline structure of cellulose fibrils for MBSM powders. Vapour pressure during drying also causes stresses within fibres resulting in enhancing the already inflicted deformation/defects in fibre walls occurred during hammer milling^36^. Drying also increases the microfibril angle^37^ which may thus influence deformation like dislocations of the already weaken cellulose fibre walls. The impact of hammer mill together with the added effect of drying stress most likely resulted in observed enhanced fibre wall deformation in dried HMP with possible influence on the crystalline structure of cellulose fibrils.

### Extractive content in wood powders

The extractive content analysis showed that non-dried powders had higher extractives than dried powders (Fig. S3). The non-dried powders of GMP and FMP showed a more or less similar amount of determined extractive content. Non-dried and dried MBSM powders (DMP) obtained from dried wood showed lower content of extractives except oxidised resin acids compared to non-dried powders obtained from green (GMP) and fibre saturation (FMP) wood and HMP. The GMP was obtained from green wood and FMP was generated from wood having moisture content at fibre saturation level. It seems that the two conditions did not influence differently on extractives causing a similar amount of extractive content for both types. Further, non-dried and dried DMP and HMP showed lower extractive content compared to non-dried and dried GMP and FMP. DMP and HMP were produced from dried woods and chips, respectively. The heat during dying may influence extractive content and quality^38^. The reduction in extractive content depends on the temperature and time. The reduction increases with the increase in temperature and time^39,40^. The powders were dried at 105 ^º^C for 16–20 h. Thus, the drying condition used should influence extractive content leading to less amounts found with powders from dried wood and chips in comparison to the powders from green and fibre saturated wood.

### Micro-distribution of extractives in raw wood material

#### Triglycerides

**Fig. 3:**
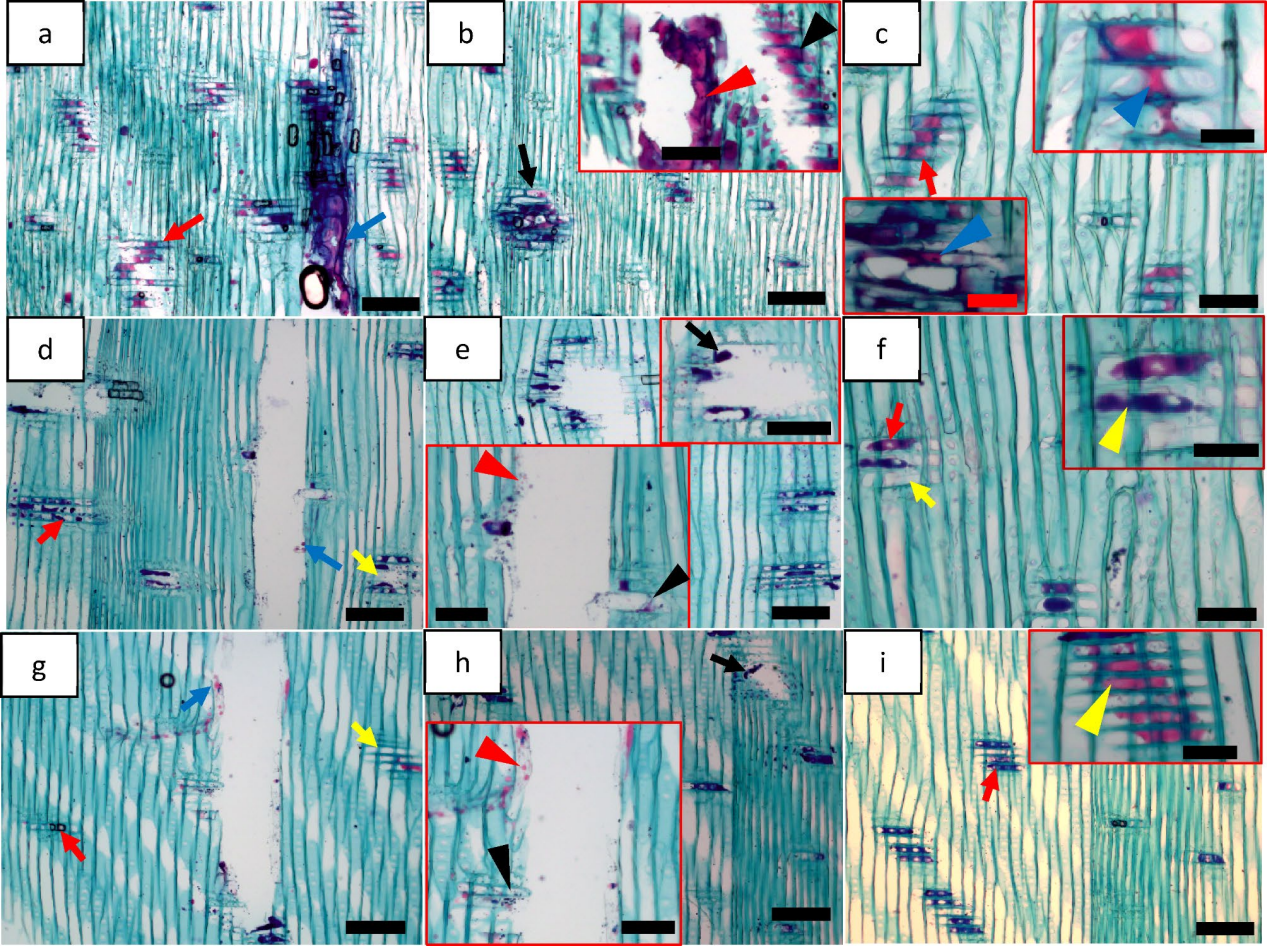
Localization of neutral triglycerides (red/pink staining) in the cellular system of pine wood (radial longitudinal sections) using Nile blue (NB) showing presence and micro-distribution of native triglycerides for green wood (**a**,** b** and **c**), fibre saturation wood (**d**,** e** and **f**) and dry wood (**g**,** h** and **i**). Bars: a, b, b (inset), d, e, e (inset top right) g, h, h (inset), i, 100 μm; c, c (inset bottom left), f, 50 μm; c (inset top right), e (inset left bottom), i (inset), 70 μm; f (inset), 30 μm.

As triglycerides are one of the major lipophilic extractives in pine wood, cytochemical staining using Nile blue (NB) was performed on triglycerides to gain information on their micromorphological features in the native state. NB stains neutral triglycerides red/pink^41^ and current results showed their presence in ray parenchyma (red arrows, Fig. 3a, c, d, f, g and i) and epithelial cells of both vertical- (blue arrows, Fig. 3a, d and g) and horizontal resin canals (black arrows, Fig. 3b, e (inset top right) and h) for green, fibre saturation and dry wood. Triglycerides were observed located in the corner of the cell lumen of both ray parenchyma (blue arrowhead; inset top right in Fig. 3c) and epithelial cells (blue arrowhead; inset bottom left in Fig. 3c) of green wood but they were dispersed in the cell lumen for fibre saturation wood (yellow arrowhead; inset top right in Fig. 3f) and dried wood (yellow arrowhead; inset top right in Fig. 3i). Furthermore, they were spread out across the cell lumen of epithelial cell (red arrowhead; inset top right in Fig. 3b) and ray parenchyma cell of adjacent resin canals (black arrowhead; inset top right in Fig. 3b) were visible for green wood, while fewer red droplets were observed in epithelial cell (red arrowhead; inset bottom left in Fig. 3e) and ray parenchyma cell of adjacent resin canals (black arrowhead; inset bottom left in Fig. 3e) for fibre saturation wood. For dried wood, fewer droplets were also observed in epithelial cell (red arrowhead; inset bottom left in Fig. 3h) and ray parenchyma cell (black arrowhead; inset bottom left in Fig. 3h) of adjacent resin canals. Observation indicate a difference in the micro-distribution and micro-morphological features of neutral fats/triglycerides presence between dried and non-dried wood. There were also non-stained ray parenchyma observed for dried wood having moisture content at fibre saturation level (yellow arrow, Fig. 3d and f) and dried wood below fibre saturation level (yellow arrow, Fig. 3g). However, almost all of the ray parenchyma and epithelial cells showed the presence of triglycerides for green wood (Fig. 3a, b and c). Heat reduces the extractive content in wood^38–40^, and it is thus likely responsible for the weak/no staining result indicating less amount of triglycerides in dried wood.

#### Lipids

**Fig. 4:**
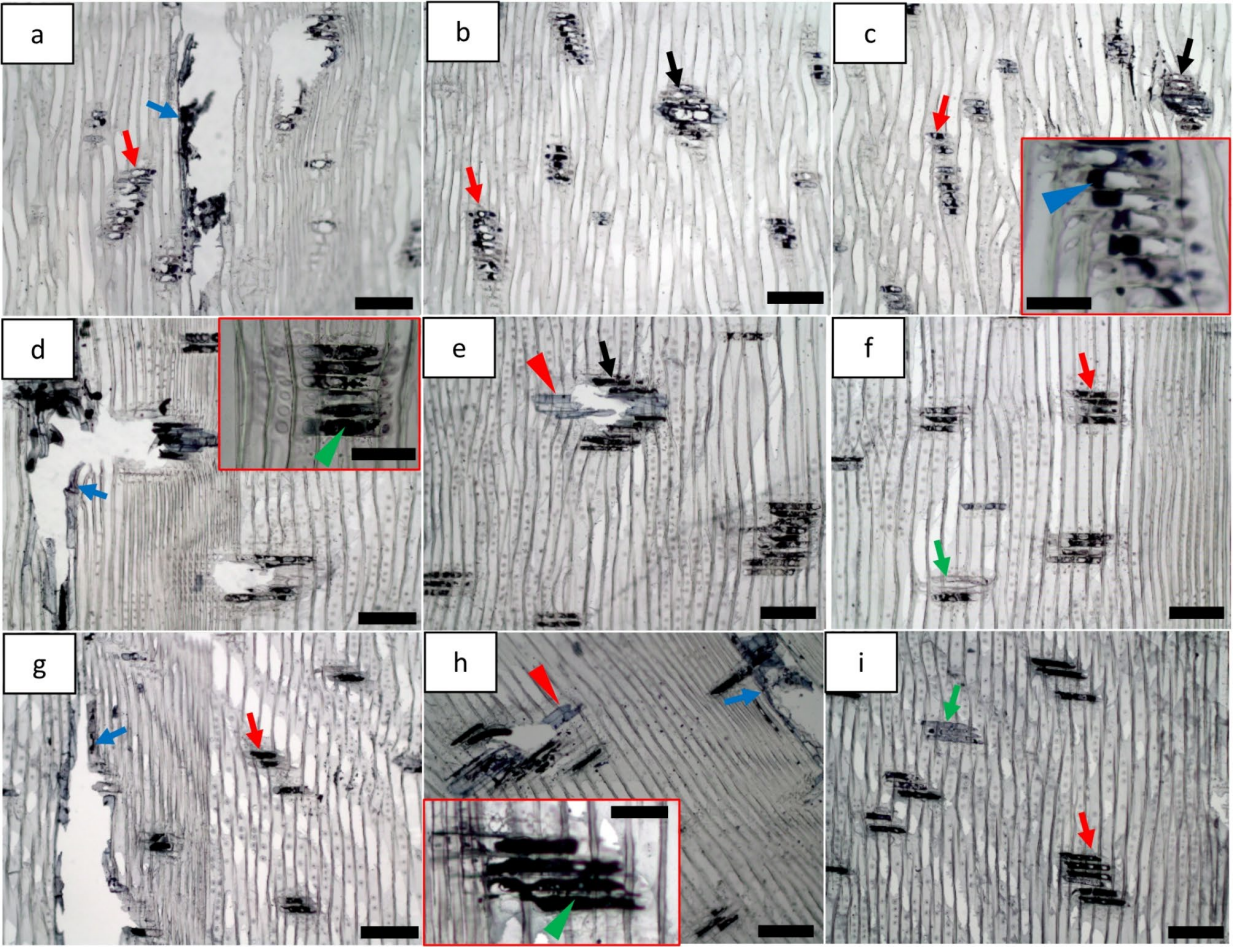
Localization of lipids (i.e. fats/free fatty acids) using Sudan black B (SB) for green wood (**a**,** b** and **c**), fibre saturation wood (**d**,** e** and **f**), and dry wood (**g**,** h** and **i**). Bars: a, b, c, d, d (inset), e, f, g, h, i, 100 μm; c (inset), 50 μm; h (inset), 70 μm.

Sudan black B (SB) method stains all lipids including fats and free fatty acids (FA) blue-black. SB stained ray parenchyma (red arrow) and epithelial cells lining resin canals (both vertical (blue arrow) and horizontal (black arrow)) blue-black revealing the presence of fats/free FA (Fig. 4a-i) for green, fibre saturation and dry wood. They were present in the corner of parenchyma (blue arrowhead; inset bottom right in Fig. 4c) for green wood but dispersed inside the lumina of parenchyma for fibre saturation wood (green arrowhead; inset top right in Fig. 4d) and dried wood (green arrowhead; inset bottom left in Fig. 4h). Similar to triglyceride micro-morphology, there was a difference in lipids micro-distribution in never dried green wood. Observations indicated that lipids were present in almost all of the ray parenchyma and epithelial cells for green wood (Fig. 4a, b and c). Nevertheless, there were sometimes non-stained/weak-stained ray parenchyma (green arrow) and epithelial cells (red arrowhead) observed for dried wood having moisture content at fibre saturation level (Fig. 4e and f) and dried wood below fibre saturation level (Fig. 4h and i) indicating less amount of lipids. It is previously shown that heat treatment can cause reduction in extractive content of wood^38–40^ and the results of the present study also support the similar effect of drying on lipids.

#### Unsaturated fats

**Fig. 5:**
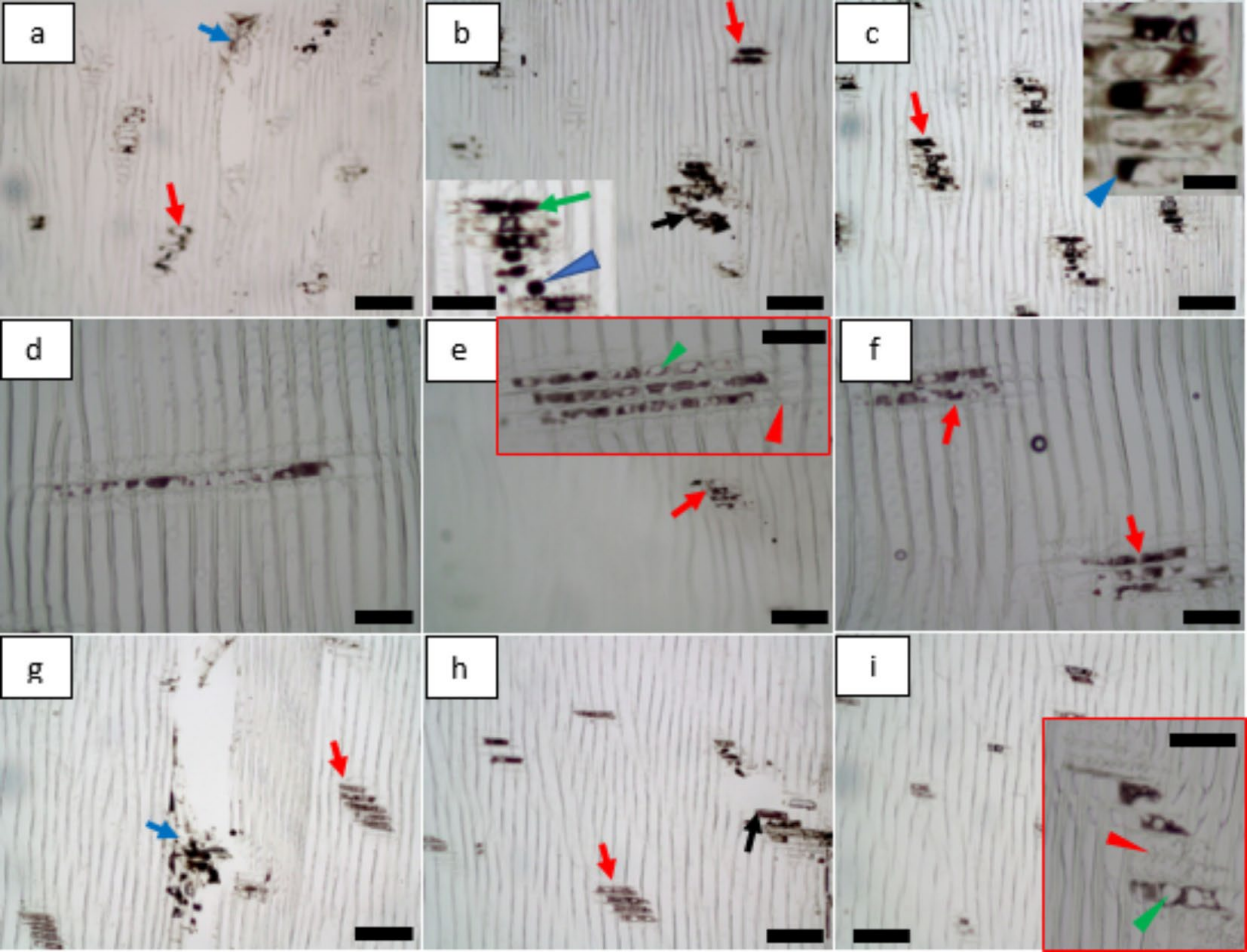
Localization of unsaturated fats using osmium tetroxide for green pine wood (**a**,** b** and **c**), fibre saturation wood (**d**,** e** and **f**), and dry wood (**g**,** h** and **i**). Bars: a, b, b (inset), c, c (inset), d, e, f, g, h, i, i (inset), 100 μm; e (inset), 70 μm.

The presence and distribution of lipids containing double bonds (i.e. unsaturated fats/FAs) can be studies using osmium tetroxide which stains unsaturated fats black^41^. After staining with osmium tetroxide, contents of ray parenchyma (red arrow) and epithelial cells of resin canals (vertical (blue arrow), and horizontal (black arrow)) were stained black revealing the presence of unsaturated fats (Fig. 5). They were observed in the corner of the cells for green wood (blue arrowhead; inset top right in Fig. 5c) but distributed within the cells for dried wood (Fig. 5d, e, f, g, h and i). Similar to other lipophilic extractives shown above, unsaturated fats were also found as large black staining droplets (blue arrowhead; inset bottom left in Fig. 5b) in the cell lumen or spread out in across the ray cells (green arrow; inset bottom left in Fig. 5b) for green wood. Thus, unsaturated fats were distributed differently for never dried green wood compared to dried wood. Although almost all of the ray parenchyma and epithelial cells were observed to contain unsaturated fats for green wood (Fig. 5a, b and c), it was not the case for dried wood. There were some ray parenchyma cells that were observed unstained for dried wood having moisture content at fibre saturation level (red arrowhead; inset top right in Fig. 5e) and dried wood below fibre saturation level (red arrowhead; inset bottom right in Fig. 5i). Further, they distributed unevenly in the parenchyma cell lumen giving a weak staining response for dried wood having moisture content at fibre saturation level (green arrowhead; inset top right in Fig. 5e) and dried wood below fibre saturation level (green arrowhead; inset bottom right in Fig. 5i). This indicated a comparatively low amount of unsaturated fats in dried wood. As mentioned above, heat treatment reduces the extractive content in wood^38–40^ and our observation on the presence/distribution of relatively less amount of unsaturated fats in dried wood is in agreement with those previous findings.

### Effect of milling type and parameters and wood powders drying on extractive distribution

#### Triglycerides

**Fig. 6:**
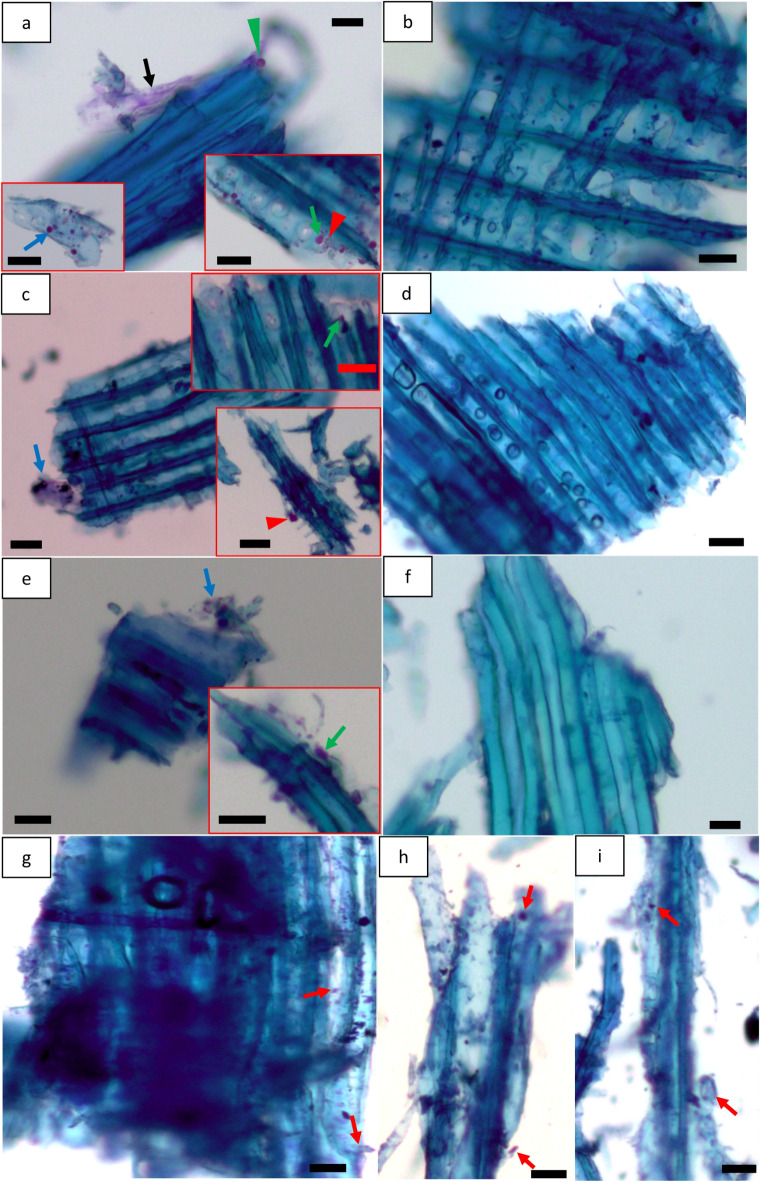
Localization of neutral triglycerides (red/pink staining) in the cellular system of pine wood powder using Nile blue (NB) for MBSM (**a-f**) and hammer mill (HMP) powders (**g**,** h**): (**a**,** c**,** e** and **g**) show non-dried MBSM and HM powders where (**a**) is green wood powder (GMP); (**c**) for fibre saturation wood powder (FMP); (**e**) for dry wood powder (DMP); and (**g**) for hammer mill powder (HMP). (**b**,** d**,** f**,** h** and **i**) show dried MBSM and HM powders where (**b**) for green wood MBSM powder (GMP), (**d**) for fibre saturation wood MBSM powder (FMP); (**f**) for dry wood MBSM powder (DMP); and (**h**,** i**) for hammer mill powder (HMP). Bars: a, c, e (inset), f, h, 30 μm; a (inset bottom left), c (inset bottom right), 100 μm; a (inset bottom right), b, i, 50 μm; c (inset top right), d, e, g, 70 μm.

Previous studies show extractives micro-distribution and behaviour during pulping processes using histochemical staining techniques^41,42^. Similar observation was also observed with milled powders in the present study. The powders obtained from MBSM (Fig. 6a-f) and hammer mill (Fig. 6g-i) showed different micro-morphological features of extractives and their micro-distribution. There was also a difference among the different types of MBSM powders. Red/pink staining was observed with intact parenchyma cells present in particles for non-dried GMP (black arrow, Fig. 6a) and FMP (red arrowhead; inset bottom right in Fig. 6c) and with intact epithelial cells for non-dried DMP (green arrow; inset bottom right in Fig. 6e). They were observed as small globules redistributed over fibres for GMP (green arrowhead, Fig. 6a). They were also distributed as small (red arrowhead; inset bottom right in Fig. 6a) and large globules (green arrow; inset bottom right in Fig. 6a) in bordered pits for non-dried GMP while they were as small globules in bordered pits for non-dried FMP (green arrow; inset top right in Fig. 6c). Triglycerides were also visible as large and small globules redistributed over the surfaces of fibres/broken fibre parts for non-dried GMP (blue arrow; inset bottom left in Fig. 6a), FMP (blue arrow, Fig. 6c) and DMP (blue arrow, Fig. 6e). However, dried MBSM powders showed more or less clear surface (Fig. 6b, d and f). They were, on the other hand, distributed over the particle surface for non-dried (red arrow, Fig. 6g) and dried (red arrows, Fig. 6h and i) HMP. The staining showed comparatively strong response for the non-dried MBSM powder (i.e., GMP) obtained from green wood (Fig. 6a) compared to non-dried MBSM powders (i.e., FMP and GMP) obtained from dried wood (Fig. 6c and e) and hammer mill (Fig. 6g-i) powders. Unlike HMP, MBSM works as cutting wood logs into smaller particles with intact parenchyma and/or unbroken cells that can still retain their extractives (e.g. triglycerides) inside the cells. However, the impact and shearing force acting on wood cellular elements during HMP process can destroy the parenchyma and epithelial cells leading to liberating triglyceride out of the cells and spreading over the particle surface. The drying reduces extractive content and consequently may be responsible for weak staining response and small globules for the MBSM powders obtained from dried wood and hammer mill powders. It may also result in more or less absence of globules on the particles surface of dried MBSM powders.

#### Lipids

**Fig. 7:**
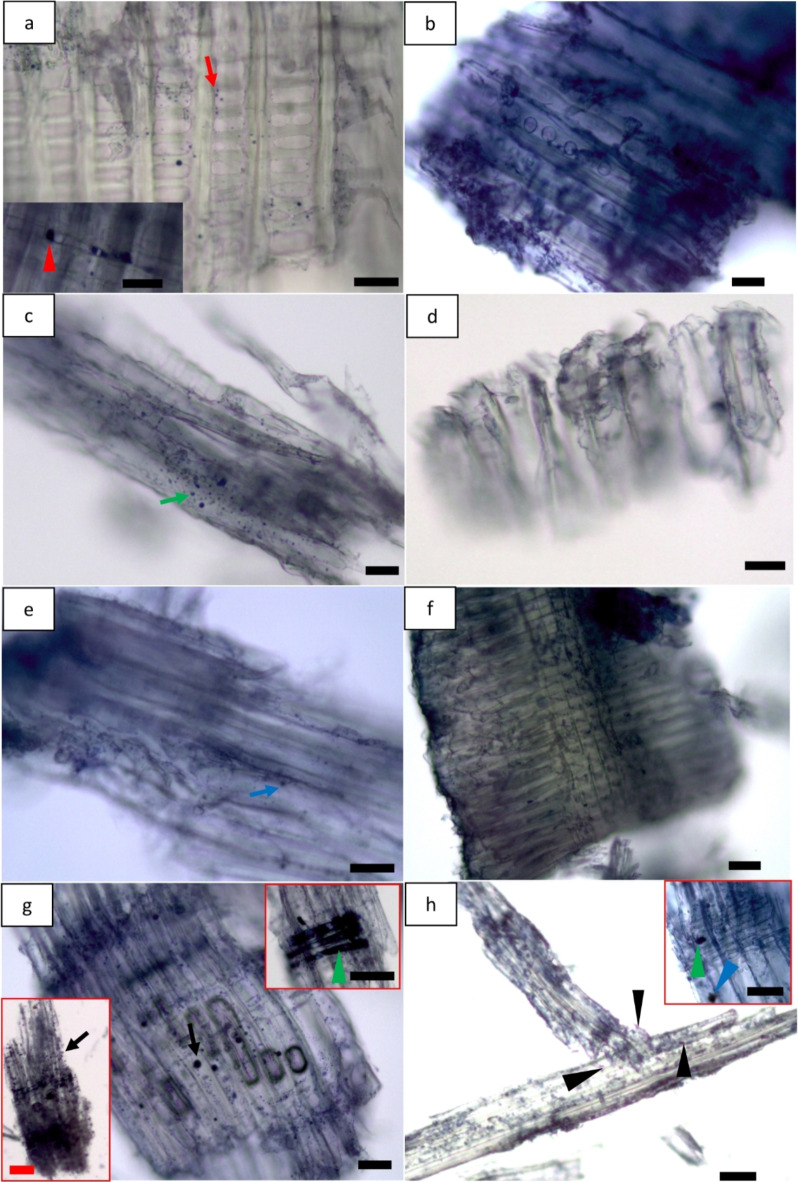
Localization of lipids (i.e. fats/free fatty acids) of pine wood powders using Sudan black B (SB) for MBSM (**a-f**) and hammer mill (HMP) powders (**g**,** h**): (**a**,** c**,** e** and **g**) show non-dried MBSM and HM powders where (**a**) is green wood powder (GMP); (**c**) for fibre saturation wood powder (FMP); (**e**) for dry wood powder (DMP); and (**g**) for hammer mill powder (HMP). (**b**,** d**,** f** and **h**) show dried MBSM and HM powders where (**b**) for green wood MBSM powder (GMP), (**d**) for fibre saturation wood MBSM powder (FMP); (**f**) for dry wood MBSM powder (DMP); and (**h**) for hammer mill powder (HMP). Bars: a, e, 20 μm; a (inset), d, 50 μm; b, c, f, g, 30 μm; g (inset top right), g (inset bottom left), h, h (inset), 100 μm.

SB stained parenchyma cell lumen in powder particles black (red arrowhead, inset bottom left, Fig. 7a) indicating presence of lipids (i.e. fats/free fatty acids (FAs)) in the cell corner or lipids were redistributed as small globules with less quantity over the particle surface for GMP (red arrow, Fig. 7a). They were also redistributed as small globules and in small quantity for FMP (green arrow, Fig. 7c) and DMP (blue arrow, Fig. 7e). However, dried MBSM powders (i.e., GMP (Fig. 7b), FMP (Fig. 7d) and DMP (Fig. 7f)) showed more or less clear surface. They were redistributed as large globules and comparatively in large quantity on the particle surface for non-dried HMP (black arrows, Fig. 7g) but existed as small globules in large quantity (black arrowhead, Fig. 7h) and large globules in small quantity (blue arrowhead; inset top right top in Fig. 7h) on the particle surface for dried HMP. They were also found as dispersed in the ray parenchyma cell for non-dried (green arrowhead; inset top right in Fig. 7g) and dried (green arrowhead, inset top right in Fig. 7h) HMP. As discussed earlier, MBSM with cutting force may be responsible for unbroken parenchyma cells and thereby retaining lipids inside the cells. For hammer mill, the impact and shearing force caused destruction of some parenchyma/epithelial cells resulting in spreading lipids over the particle surface including dispersion in the cell lumen. As discussed earlier drying may cause less amount of lipids over particles as observed for powders from dried wood and the dried powders.

#### Unsaturated fats

**Fig. 8:**
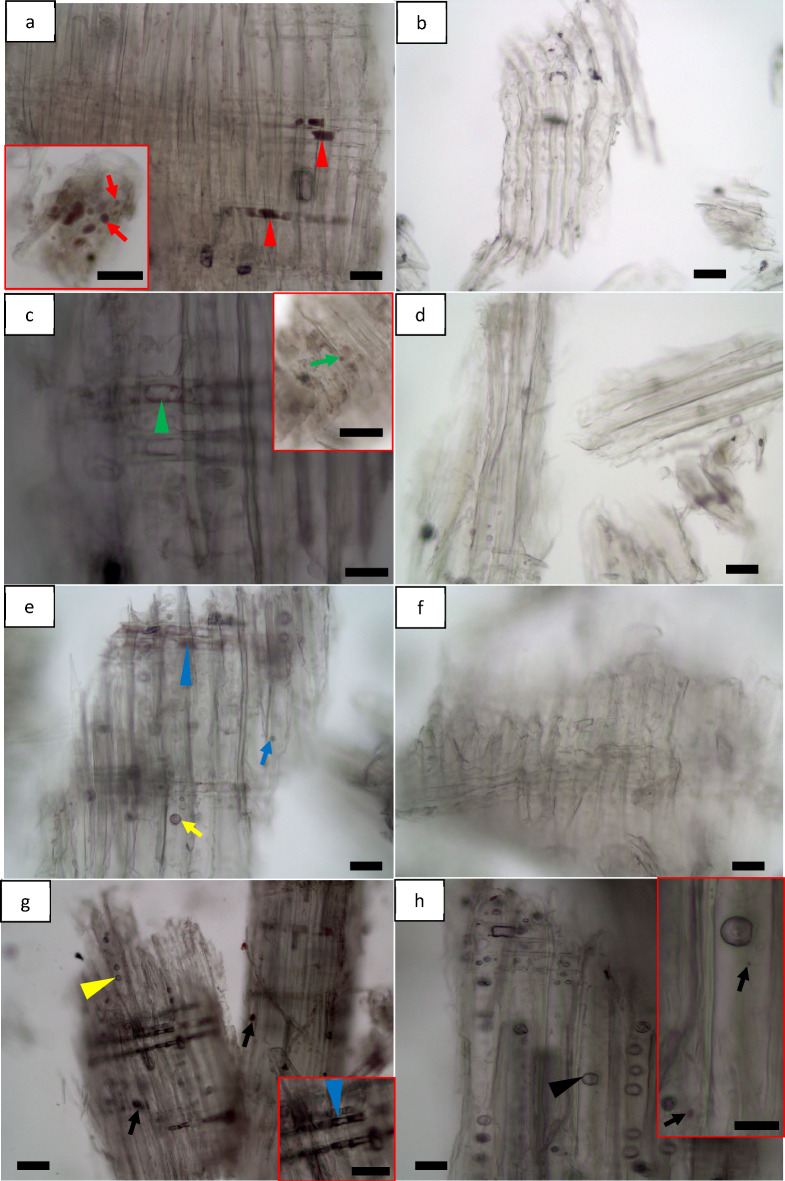
Localization of unsaturated fats in pine wood powder using osmium tetroxide for MBSM (**a-f**) and hammer mill (HMP) powders (**g**,** h**): (**a**,** c**,** e** and **g**) show non-dried MBSM and HM powders where (**a**) is green wood powder (GMP); (**c**) for fibre saturation wood powder (FMP); (**e**) for dry wood powder (DMP); and (**g**) for hammer mill powder (HMP). (**b**,** d**,** f** and **h**) show dried MBSM and HM powders where (**b**) for green wood MBSM powder (GMP); (**d**) for fibre saturation wood MBSM powder (FMP); (**f**) for dry wood MBSM powder (DMP); and (**h**) for hammer mill powder (HMP). Bars: a, a (inset), b, d, e, f, h, 30 μm; c, 20 μm; c (inset), g (inset), 100 μm; g, h (inset), 50 μm.

Osmium tetroxide stained cell lumen of parenchyma black for GMP (red arrowheads, Fig. 8a), FMP (green arrowhead, Fig. 8c) and DMP (blue arrowhead, Fig. 8e) of non-dried MBSM powders indicating retaining of unsaturated fats/free FAs inside the cells. They were also found to distribute as small and large globules (red arrows; inset bottom left in Fig. 8a) on the particle surface for non-dried GMP. They were redistributed as comparatively small globules of unsaturated fats on particle surface (green arrow; inset top right in Fig. 8c and blue arrow, Fig. 8e) as well as weakly stained globules present in parenchyma cells (green arrowhead, Fig. 8c and blue arrowhead, Fig. 8e) for non-dried FMP and DMP. They were also observed in bordered pit (yellow arrow, Fig. 8e) for non-dried DMP. For hammer mill powders, they were distributed in bordered pit (yellow arrowhead, Fig. 8g), as large globules on the particle surface (black arrows, Fig. 8g) and dispersed in the parenchyma cell (blue arrowhead; inset bottom right in Fig. 8g) for non-dried HMP. For dried HMP, these were still observed in the bordered pit (black arrowhead, Fig. 8h) and on the surface as small globules (black arrows; inset top right in Fig. 8h). However, dried MBSM powders (i.e., GMP, FMP and DMP) showed more or less free from globules indicating the absence of unsaturated fats (Fig. 8b, d and f). As previously discussed, milling mechanisms and drying effect presumably responsible for the observed differences in extractives micro-distribution and micro-morphological features of unsaturated fats. The effect of drying of wood and wood powder on the other extractives discussed above was also observed for unsaturated fats.

The results of this study provide a foundation for future research into specific wood powder applications and the influence of milling processes. As with all new technologies, it is difficult to predict the range of future applications, which may immerge for MBSM powders.

Powders produced from green wood may be especially attractive for biochemical applications where, bases on the results herein, the original extractive profile of the wood is mostly intact. Due to their fine (and narrow) particle size distribution and extractive location retention, milling green biomass will likely enhance the efficiency of bioconversion, for example owning simply to greater surface area and porosity^13^, and particle size distribution has been shown to be a major yield factor in hydrolysis^43^. This same enhanced reactivity of MBSM powder will likely lead to increased conversion rates in thermal processes (for dry powders).

For products made from densified wood powders, such as fuel pellets and fibre boards, it is conceivable that the differences in extractive profile, may affect binding and adhesion mechanisms. For example, greater extractive content was claimed to negatively affect fuel pellet densification^44^, yet as the study used hammer milled powder (which have greater extractive distribution on the particle surface), the extractive locations in the powder were not considered. Hammer mill powders, on the other hand, may be beneficial for conversion of cellulose to glucose since these particles exhibit a less crystalline structure.

## Conclusions

The morphology and native extractives micro-distribution of wood powders obtained from a new MBSM technology were analysed. These were compared with the conventional powders produced by a hammer mill. The study revealed that both dried and non-dried MBSM powders had a smooth surface and wood fibres with less structural deformation/defects of their cell walls. In contrast, hammer mill powders had a rough surface with fibres showing some cell wall deformations (e.g. dislocation). Extractives were observed mostly intact within cell lumen for non-dried MBSM powders, and most of the dried MBSM powders showed a clear surface. Non-dried and dried hammer mill powders, on the other hand, were covered with extractives and they were also dispersed in the cell lumen. Original wood samples, however, had extractives in the cell lumen. It is interesting to note that extractives in non-dired MBSM powders were mostly found to be in locations and in form as they were in the original wood samples. These findings indicate that morphology and extractive distribution in powders, as produced by hammer mills versus multi-blade shaft mills, may indeed influence their application in downstream processes.

## Electronic supplementary material

Below is the link to the electronic supplementary material.

Supplementary Material 1

## Data Availability

The datasets used and/or analysed during the current study are available from the corresponding author on reasonable request.

## Abbreviations

BET: Brunauer-Emmett-Teller
BTC: Biomass technology centre
DMP: MBSM powder from dry wood
FAs: Fatty acids
FMP: MBSM powder from wood at fibre saturation point
GMP: MBSM powder from green wood
HMP: Hammer mill powder
LM: Light microscopy
MBSM: Multi-blade shaft mill
MC: Moisture content
MFA: Microfibril angle
NB: Nile blue
PLM: Polarized light microscopy
SB: Sudan black B
WPC: Wood plastic composites
