# Supplementary material for: Micromorphology and native extractive behaviour of wood powder
Source: Sci Rep. 2024 Oct 26;14:25548. doi: 10.1038/s41598-024-75716-3 (PMC11513130; doi:10.1038/s41598-024-75716-3)
Supplement: Supplementary file 1 — Supplementary Material 1 [file 41598_2024_75716_MOESM1_ESM.pdf]

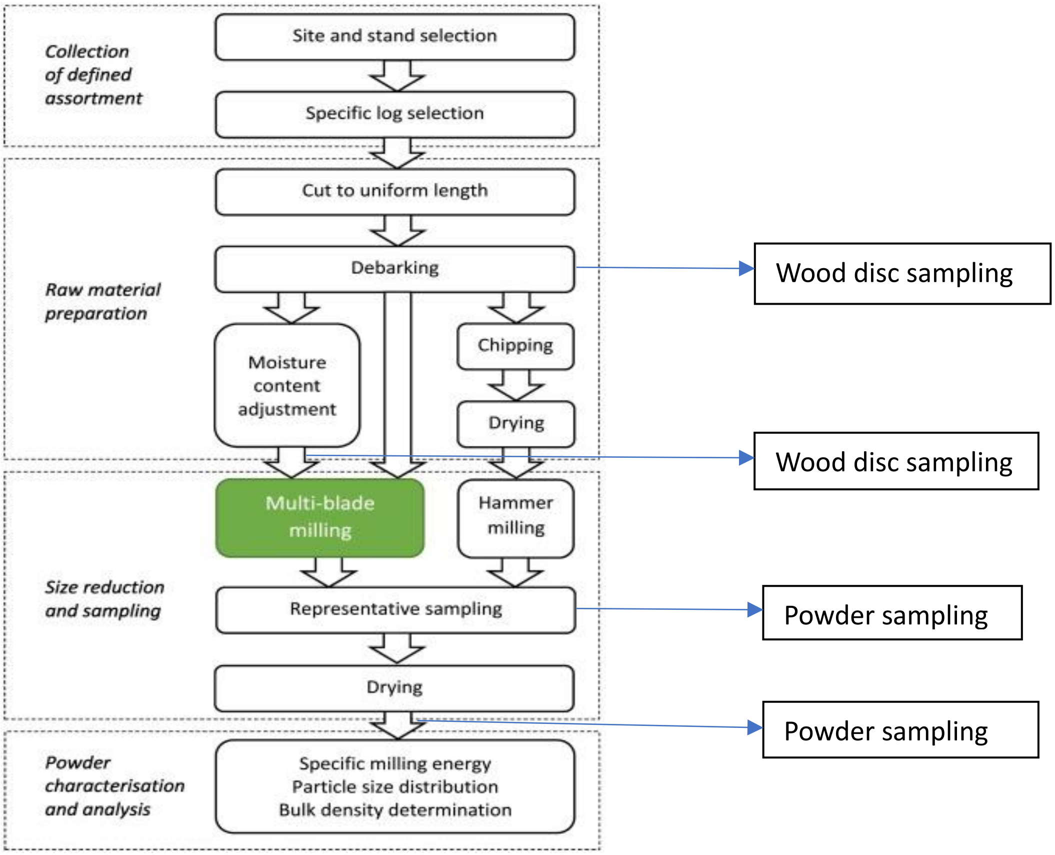


Fig. S1: Sampling stages of wood discs and powders.

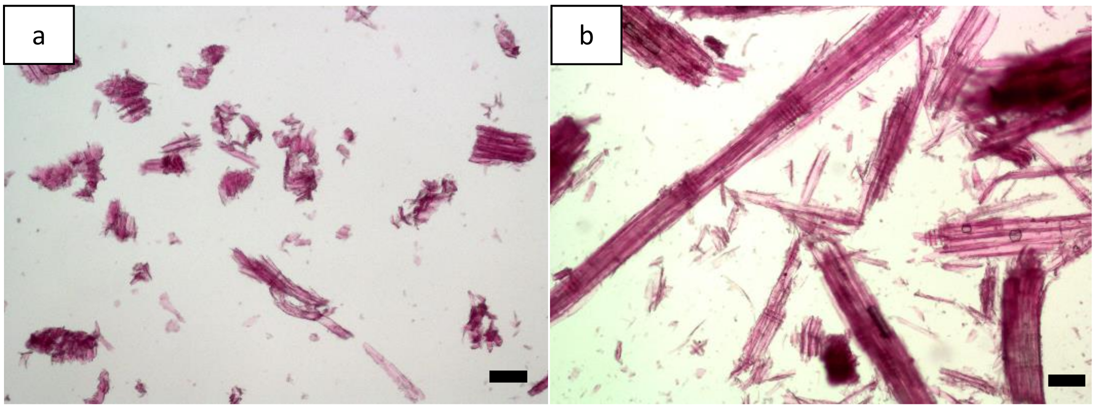


Fig. S2. Physical appearance for dry wood MBSM powder (DMP; a) and hammer mill powder (HMP; b). Bars: a, b, 100 µm.

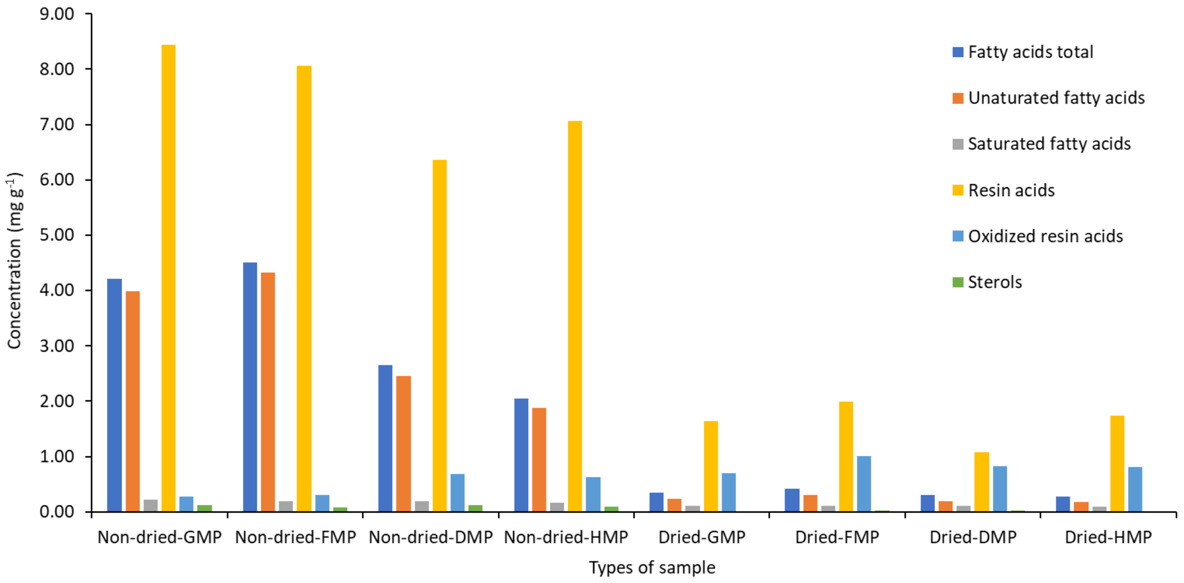


Fig. S3. Extractive content of non-dried and dried green wood MBSM powder (GMP), fibre saturation wood MBSM powder (FMP), dry wood MBSM powder (DMP) and hammer mill powder (HMP).

Table S1: An overview of the processing of wood powder sample and properties.

| Sample name | Milling parameters [33] | | | Powder properties [13,33] | | | |
|---|---|---|---|---|---|---|---|
| | Wood moisture content (%) | Feeding speed (m $min^{-1}$) | Blade speed (m $s^{-1}$) | Particle <1 mm (%) | Bulk density (kg $m^{-3}$ DM ) | BET specific surface area ($m^2$ $g^{-1}$) | Pore volume ($cm^3$ $g^{-1}$) |
| Green Wood MBSM powder (GMP) | 42.0 | 1.32 | 72 | 94.0 | 138 | 1.378 | 0.002759 |
| Fibre saturation wood MBSM powder (FMP) | 30.7 | 1.86 | 62 | 88.5 | 174.1 | - | - |
| Dry Wood MBSM powder (DMP) | 13.0 | 1.38 | 72 | 92.1 | 264.4 | 1.32 | 0.001035 |
| Dry wood chip Hammer mill powder (HM) | - | - | - | 92.9 | 219.3 | 1.06 | 0.001037 |
